# Construction of the machine learning-based live birth prediction models for the first in vitro fertilization pregnant women

**DOI:** 10.1186/s12884-023-05775-3

**Published:** 2023-06-27

**Authors:** Xiaoyan Liu, Zhiyun Chen, Yanqin Ji

**Affiliations:** 1 Reproductive Medicine Center, Huizhou Municipal Central Hospital, Huizhou, 516001 P.R. China; 2 Obstetrics and Gynecology, Huizhou Municipal Central Hospital, Xiapu Branch, No. 8 Hengjiang 4Th Road, Huizhou, 516001 P.R. China

**Keywords:** Machine learning-based, Live birth, Prediction models, In vitro fertilization

## Abstract

**Background:**

This study was to conduct prediction models based on parameters before and after the first cycle, respectively, to predict live births in women who received fresh or frozen in vitro fertilization (IVF) or intracytoplasmic sperm injection (ICSI) for the first time.

**Methods:**

This retrospective cohort study population consisted of 1,857 women undergoing the IVF cycle from 2019 to 2021 at Huizhou Municipal Central Hospital. The data between 2019 and 2020 were completely randomly divided into a training set and a validation set (8:2). The data from 2021 was used as the testing set, and the bootstrap validation was carried out by extracting 30% of the data for 200 times on the total data set. In the training set, variables are divided into those before the first cycle and after the first cycle. Then, predictive factors before the first cycle and after the first cycle were screened. Based on the predictive factors, four supervised machine learning algorithms were respectively considered to build the predictive models: logistic regression (LR), random forest (RF), extreme gradient boosting (XGBoost), and light gradient boosting machine (LGBM). The performances of the prediction models were evaluated by the area under the receiver operator characteristic curve (AUC), sensitivity, specificity, positive predictive value (PPV), negative predictive value (NPV), and accuracy.

**Results:**

Totally, 851 women (45.83%) had a live birth. The LGBM model showed a robust performance in predicting live birth before the first cycle, with AUC being 0.678 [95% confidence interval (CI): 0.651 to 0.706] in the training set, 0.612 (95% CI: 0.553 to 0.670) in the validation set, 0.634 (95% CI: 0.511 to 0.758) in the testing set, and 0.670 (95% CI: 0.626 to 0.715) in the bootstrap validation. The AUC value in the training set, validation set, testing set, and bootstrap of LGBM to predict live birth after the first cycle was 0.841 (95% CI: 0.821 to 0.861), 0.816 (95% CI: 0.773 to 0.859), 0.835 (95% CI: 0.743 to 0.926), and 0.839 (95% CI: 0.806 to 0.871), respectively.

**Conclusion:**

The LGBM model based on the predictive factors before and after the first cycle for live birth in women showed a good predictive performance. Therefore, it may assist fertility specialists and patients to adjust the appropriate treatment strategy.

## Background

Infertility is defined as the failure of husband and wife to get pregnant after 12 months of regular sexual intercourse and without using any contraceptive method [1]. It is estimated that 8–12% of couples of reproductive ages worldwide face this problem, affecting family well-being and social stability worldwide [2]. Thereby, the demand for in vitro fertilization (IVF) and intracytoplasmic sperm injection (ICSI) is increasing, with over 8 million babies born through IVF or other assisted reproductive technology treatments since the world’s first in 1978 [3]. Although the success rate of assisted reproductive technology increases with newer techniques, IVF does not guarantee success in fertilization [4]. Considering the high financial burden and inconclusive health risk of IVF [5], it is necessary to explore the factors related to the live birth of women with IVF and construct prediction models to predict the live birth rate of IVF pregnant women for patients counseling and shaping expectations.

Previous studies have reported that age, duration of infertility, previous live birth, previous miscarriage, previous abortion, and type of infertility were associated with the IVF outcome [6, 7]. Using these parameters that can be obtained before the IVF cycle to evaluate live births may improve the treatment efficiency of assisted reproductive technology and reduce the waste of resources. Moreover, assisted reproductive process-related factors, such as stimulation protocols, and number of embryos transferred were also found to be related to the outcome of assisted reproductive technology [8, 9]. The outcome prediction model based on the above post-cycle factors is conducive to doctor-patient communication and can help clinicians adjust assisted reproductive planning. The traditional prediction relies on the comprehensive evaluation of the doctor based on the age of the patient and the live birth rate of the reproductive center, which is highly subjective [4]. Up till now, reliable and accurate prediction of IVF outcomes has always been an outstanding issue. Machine-learning algorithms have recently been used to predict IVF outcomes on the basis of multiple clinical variables [7]. A recent study reported that the predicted probability of live birth in IVF varies during important stages throughout the treatment [10]. However, few studies have established prediction models respectively before the first cycle and after the first cycle. Assessing outcomes before and after the first cycle is critical to provide individualized treatment for infertile patients. Moreover, previous prediction models for live birth were mostly based on fresh embryo transfer [11, 12], but did not fully consider frozen embryo transfer. In view of the development of frozen embryo transfer, it is necessary to conduct prediction models to predict live births of based on the fresh and frozen embryo transfer.

Herein, the objective of the present study is to use machine learning methods to conduct prediction based on parameters before and after the first cycle, respectively, to predict live births in women who received IVF for the first time.

## Methods

### Study design and population

In this retrospective cohort study, women undergoing IVF (including ICSI) at the Huizhou Municipal Central Hospital from 2019 to 2021 were retrospectively reviewed. Infertile women with age between 20 and 45 years who received IVF or ICSI for the first time were included in this study. The study was approved by the ethics committee of Huizhou Municipal Central Hospital (ky112022001). Patient informed consent was waived for this retrospective study by the ethics committee of Huizhou Municipal Central Hospital. All methods were carried out in accordance with relevant guidelines and regulations (Declaration of Helsinki).

### Data collection

Data were collected from the case report form (CRF), including (1) demographic data: maternal age (years), maternal body mass index (BMI, kg/m^2^); (2) before the first cycle: causes of infertility (man, women, both, and unknown), the number of pregnancies, the number of deliveries, the number of miscarriages, type of infertility (primary infertility and secondary infertility), infertility duration (years), the number of left sinus follicles (< = 7 or > 7), the number of right sinus follicles (< = 7 or > 7), basal follicle-stimulating hormone (FSH, mIU/ml), basal luteinizing hormone (LH, mIU/ml), basal prolactin (PRL, ng/ml), basal serum estradiol (E2, pg/ml), basal progesterone (P, ng/ml), anti-mullerian hormone (AMH, ng/ml); (3) after the first cycle: method of fertilizations (IVF or ICSI), type of embryos (fresh embryos and frozen embryos), gonadotropin (Gn, IU), duration of Gn (days), human chorionic gonadotropin (HCG, IU/ml), LH level on HCG day (mIU/ml), E2 level on HCG day (pg/ml), endometrial thickness (EMT) on HCG day (mm), P on HCG day (ng/ml), types of transferred embryos (blastocyst and cleavage embryo), the number of embryos transferred, the number of cleavage embryos transferred, stimulation protocols (long protocol, antagonist protocol, and other protocol), luteal phase support (cyclogest + dydrogesterone, progesterone administered intramuscularly, crinon + dydrogesterone), and endometrial preparation protocol (ovulation induction protocol cycles, natural cycles, hormone replacement treatment cycles, and no).

Primary infertility referred to the infertility of couples who have never been pregnant, while secondary infertility referred to the failure to get pregnant after the previous pregnancy. The long protocol included prolonged protocol, long protocol in the follicular phase, and long-acting long protocol; other protocols included mild stimulation protocol, progestin-primed ovarian stimulation (PPOS), and other ovulation stimulation protocol.

### Outcome

The primary outcome of this study was a live birth rate, described as the delivery of at least one live baby at or after 24 weeks gestation.

### Prediction models constructions and evaluations

The data between 2019 and 2020 were completely randomly divided into a training set (80% of the total number) and a validation set (20%). The data of 2021 was used as the testing set, and the bootstrap validation was carried out by extracting 30% of the data for 200 times on the total data set. In the training set, variables were divided into those before the first cycle and after the first cycle. Then, predictive factors before the first cycle and after the first cycle were screened based on the data in the training set. Based on the predictive factors, four supervised machine learning algorithms were respectively considered to build the prediction models: logistic regression (LR), random forest (RF), extreme gradient boosting (XGBoost), and light gradient boosting machine (LGBM). LR is a common supervised classification algorithm with a nice probabilistic interpretation [7]. The RF is a non-parametric, nonlinear statistical machine-learning approach that combines a set of decision trees into an 'ensemble' learner of multiple trees for a stronger output prediction [13]. XGBoost is a specific implementation of gradient tree boosting that produces a risk prediction model (called a strong learner) in the form of an ensemble of weak risk prediction models (weak learners), typically decision trees [14]. LGBM is an ensemble approach that combines predictions from multiple decision trees to make well-generalized final predictions [15].

The predictive performances of the prediction models were assessed by developing receiver operating characteristic (ROC) curves and calculating the areas under them (AUC), accuracy, sensitivity, specificity, positive predictive value (PPV), and negative predictive value (NPV) with a 95% confidence interval (CI).

### Statistical analysis

Mean ± standard deviation (Mean ± SD) was used to describe the distribution of normally distributed measurement data, and a t-test was applied to compare the differences between the two groups. Median and quartiles [M (Q_1_, Q_3_)] were used to describe the distribution of measurements that did not conform to normal distribution, and the Wilcoxon rank sum test was used to compare the difference between the two groups. The counting data were described by the number of cases and the composition ratio (n (%)), and the differences between groups were compared by the chi-square test. The simple deletion method was adopted to deal with the missing values in the data, then sensitivity analysis was conducted to compare the original data with the deleted data. Least absolute shrinkage and selection operator (LASSO) (R package "glmnet") with tenfold cross-validation (lambda = 0.0362126 was selected) was used to screen the predictive variables of the model. The predictive models were internally validated using bootstrapped validation, a statistical method in which multiple evolutionary trees are constructed to check model confidence by repeatedly sampling data sets.

Results were considered significant at alpha = 0.05. Statistical analysis was conducted using R version 4.2.1 (2022–06-23 ucrt) and Python 3.9.12.

## Results

### Characteristics of included participants

**Fig. 1:**
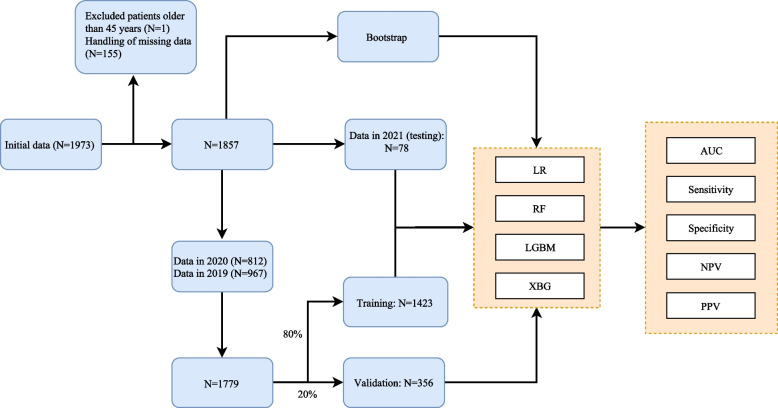
The flow diagram of data screening and participants collections

In this study, 1,857 women undergoing IVF or ICSI were analyzed, comprising 1423 women in the training set, 356 in the validation set, and 78 women in the testing set. Data selection and collection are shown in Fig. 1. Of the 1,857 women who underwent IVF or ICSI, 851 women (45.83%) had a live birth. The mean maternal age was 31.85 ± 4.51 years, with the mean maternal BMI being 21.40 (19.60, 23.60) kg/m^2^. Primary infertility and secondary infertility accounted for 57.30%, and 42.70%, respectively. The IVF and ICSI methods accounted for 81.42% and 18.58% respectively. Significant differences were found between the women who had a live birth and women without a live birth in maternal age, stimulation protocols, causes of infertility, the number of pregnancies, the number of deliveries, type of infertility, the number of left sinus follicles, the number of right sinus follicles, basal LH level, basal PR level, basal P level, AMH level, Gn, LH level on HCG day, E2 level on HCG day, the number of embryos transferred, EMT on HCG day, types of transferred embryos, and HCG (all* P* < 0.05). The baseline characteristics of the study population are summarized in Table 1.

**Table 1:** Basic characteristics of included participants

Variables	Total (*n* = 1857)	No (*n* = 1006)	Yes (*n* = 851)	Statistics	*P*
Maternal age, years, Mean ± SD	31.85 ± 4.51	32.81 ± 4.65	30.72 ± 4.06	t = 10.33	< 0.001
Fertilizations method				χ^2^ = 2.386	0.122
IVF	1512 (81.42)	832 (82.70)	680 (79.91)		
ICSI	345 (18.58)	174 (17.30)	171 (20.09)		
Stimulation protocols, n (%)				χ^2^ = 9.470	0.009
Long protocol	1336 (71.94)	696 (69.18)	640 (75.21)		
Antagonist protocol	478 (25.74)	281 (27.93)	197 (23.15)		
Other protocol	43 (2.32)	29 (2.88)	14 (1.65)		
Endometrial preparation protocol, n (%)				χ^2^ = 0.607	0.895
No	999 (53.80)	539 (53.58)	460 (54.05)		
Ovulation induction protocol cycles	61 (3.28)	31 (3.08)	30 (3.53)		
Natural cycles	234 (12.60)	125 (12.43)	109 (12.81)		
Hormone replacement treatment cycles	563 (30.32)	311 (30.91)	252 (29.61)		
Luteal phase support, n (%)				χ^2^ = 0.483	0.785
Cyclogest + dydrogesterone	1300 (70.01)	698 (69.38)	602 (70.74)		
Progesterone administered intramuscularly	328 (17.66)	183 (18.19)	145 (17.04)		
Crinon + dydrogesterone	229 (12.33)	125 (12.43)	104 (12.22)		
Causes of infertility, n (%)				χ^2^ = 11.402	0.010
Man	447 (24.07)	213 (21.17)	234 (27.50)		
Women	1063 (57.24)	607 (60.34)	456 (53.58)		
Both	217 (11.69)	117 (11.63)	100 (11.75)		
Unknown	130 (7.00)	69 (6.86)	61 (7.17)		
Maternal BMI, kg/m^2^, M (Q_1_,Q_3_)	21.40 (19.60, 23.60)	21.50 (19.70, 23.70)	21.40 (19.60, 23.40)	Z = -1.145	0.252
The number of pregnancies, M (Q_1_, Q_3_)	1.00 (0.00, 2.00)	1.00 (0.00, 2.00)	1.00 (0.00, 2.00)	Z = -2.908	0.004
The number of deliveries, M (Q_1_, Q_3_)	0.00 (0.00, 1.00)	0.00 (0.00, 1.00)	0.00 (0.00, 0.00)	Z = -3.316	< 0.001
The number of miscarriages, M (Q_1_, Q_3_)	0.00 (0.00, 1.00)	0.00 (0.00, 1.00)	0.00 (0.00, 1.00)	Z = -1.814	0.070
Type of infertility, n (%)				χ^2^ = 8.850	0.003
Primary infertility	1064 (57.30)	608 (60.44)	456 (53.58)		
Secondary infertility	793 (42.70)	398 (39.56)	395 (46.42)		
Infertility duration, years, M (Q_1_, Q_3_)	3.00 (2.00, 5.00)	3.00 (2.00, 5.00)	3.00 (2.00, 5.00)	Z = -1.513	0.130
The number of left sinus follicles, n (%)				χ^2^ = 36.892	< 0.001
< = 7	791 (42.60)	493 (49.01)	298 (35.02)		
> 7	1066 (57.40)	513 (50.99)	553 (64.98)		
The number of right sinus follicles, n (%)				χ^2^ = 17.568	< 0.001
< = 7	1096 (59.02)	638 (63.42)	458 (53.82)		
> 7	761 (40.98)	368 (36.58)	393 (46.18)		
Basal FSH, mIU/ml, M (Q_1_, Q_3_)	6.69 (5.62, 7.97)	6.75 (5.66, 8.06)	6.59 (5.56, 7.83)	Z = -1.710	0.087
Basal LH, mIU/ml, M (Q_1_, Q_3_)	5.54 (4.14, 7.39)	5.44 (4.11, 7.26)	5.68 (4.18, 7.53)	Z = 2.158	0.031
Basal PRL, ng/ml, M (Q_1_, Q_3_)	19.87 (14.37, 28.69)	19.07 (13.78, 27.76)	20.84 (14.96, 29.91)	Z = 3.047	0.002
Basal E2, pg/ml, M (Q_1_, Q_3_)	39.30 (29.00, 52.40)	39.60 (29.20, 53.00)	38.80 (28.80, 51.80)	Z = -1.455	0.146
Basal P, ng/ml, M(Q_1_,Q_3_)	0.25 (0.16, 0.38)	0.24 (0.15, 0.36)	0.26 (0.17, 0.40)	Z = 2.626	0.009
AMH, ng/ml, M (Q_1_, Q_3_)	3.00 (1.82, 4.72)	2.75 (1.63, 4.47)	3.26 (2.07, 5.11)	Z = 5.591	< 0.001
Type of embryos, n (%)				χ^2^ = 0.099	0.754
Fresh embryos	1003 (54.01)	540 (53.68)	463 (54.41)		
Frozen embryos	854 (45.99)	466 (46.32)	388 (45.59)		
The number of embryos transferred, Mean ± SD	1.68 ± 0.47	1.69 ± 0.46	1.67 ± 0.47	t = 0.82	0.415
Gn, IU, M (Q_1_, Q_3_)	2700.00 (2025.00, 3450.00)	2850.00 (2100.00, 3525.00)	2600.00 (1875.00, 3300.00)	Z = -3.755	< 0.001
duration of Gn, days, Mean ± SD	10.87 ± 2.05	10.82 ± 2.10	10.93 ± 2.00	t = -1.13	0.261
LH level on HCG day, mIU/ml, M (Q_1_, Q_3_)	1.20 (0.70, 2.08)	1.23 (0.70, 2.19)	1.14 (0.70, 1.90)	Z = -1.996	0.046
E2 level on HCG day, pg/ml, M (Q_1_, Q_3_)	2949.00 (1784.00, 4588.00)	2720.50 (1602.00, 4196.00)	3235.00 (1961.00, 4956.00)	Z = 4.844	< 0.001
The number of embryos transferred, M (Q_1_, Q_3_)	2.00 (0.00, 2.00)	2.00 (0.00, 2.00)	2.00 (0.00, 2.00)	Z = -2.507	0.012
EMT on HCG day, mm, Mean ± SD	10.72 ± 2.41	10.54 ± 2.48	10.93 ± 2.31	t = -3.45	< 0.001
P on HCG day, ng/ml, M (Q_1_, Q_3_)	0.75 (0.47, 1.19)	0.74 (0.46, 1.17)	0.78 (0.49, 1.20)	Z = 1.805	0.071
Types of transferred embryos, n (%)				χ^2^ = 14.478	< 0.001
Blastocyst	1314 (70.76)	749 (74.45)	565 (66.39)		
Cleavage embryo	543 (29.24)	257 (25.55)	286 (33.61)		
HCG, n (%)				χ^2^ = 588.084	< 0.001
< 5	509 (27.41)	508 (50.50)	1 (0.12)		
> = 5	1348 (72.59)	498 (49.50)	850 (99.88)		

*SD* standard deviation, *M* Median, *Q*_*1*_ 1st Quartile, *Q*_*3*_ 3st Quartile, *BMI* body mass index, *IVF* in vitro fertilization, *ICSI* intracytoplasmic sperm injection, *FSH* follicle-stimulating hormone, *LH* luteinizing hormone, *PRL* prolactin, *E2* serum estradiol, *T* testosterone, *P* progesterone, *AMH* anti-mullerian hormone, *Gn* gonadotropin, *EMT* endometrial thickness, *HCG* human chorionic gonadotophin, *t* T-test, *Z* Wilcoxon rank sum test; χ^2^: chi-square test

### Identification of predictive factors for live birth in women who received IVF or ICSI for the first time

**Fig. 2:**
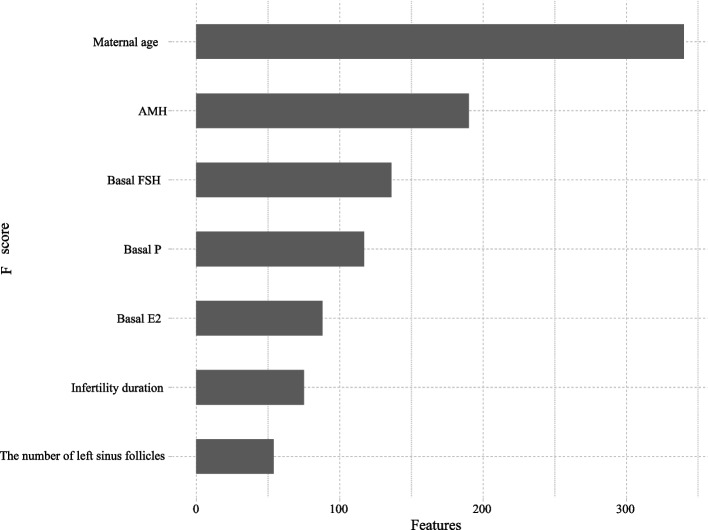
The feature importance of the predictive factors before the first cycle

The predictive factors before the first cycle included maternal age, AMH, basal FSH, basal P, basal E2, infertility duration, and the number of left sinus follicles. Identification of the predictive factors before the first cycle is shown in Fig. 2.

**Fig. 3:**
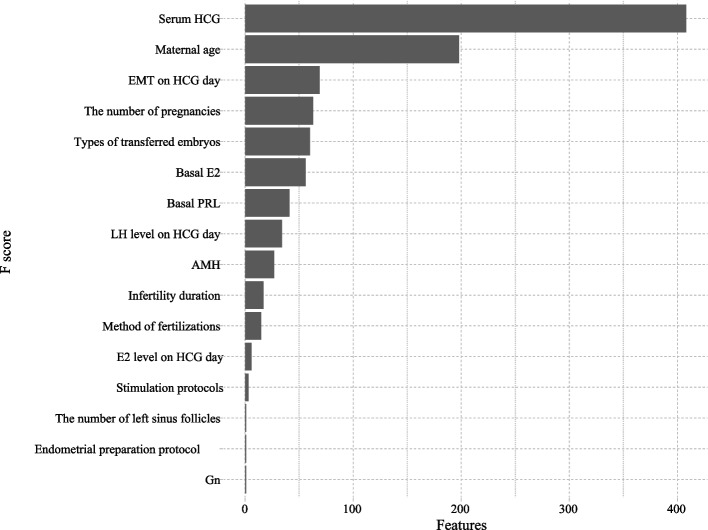
The feature importance of the predictive factors before and after the first cycle

Maternal age, serum HCG level, EMT on HCG day, the number of pregnancies, types of transferred embryos, basal E2, basal PRL, LH level on HCG day, AMH, infertility duration, method of fertilizations, E2 level on HCG day, the number of left sinus follicles, stimulation protocols, endometrial preparation protocol, and Gn were identified as the predictive factors after the first cycle. Figure 3 depicts the identification of the predictive factor after the first cycle.

### Construction and predictive performance evaluation of the predictive models for live birth in women who received IVF or ICSI for the first time

Based on the predictive factors before the first cycle, four predictive models: LR, RF, LGBM, and XGBoost were conducted. The AUC value of RF, LGBM, XGBoost, and LR, was 0.721 [95% confidence interval (CI): 0.695 to 0.748], 0.678 (95% CI: 0.651 to 0.706), 0.806 (95% CI: 0.784 to 0.829), and 0.641 (95% CI: 0.613–0.670), respectively, in the training set and 0.614 (95% CI: 0.555 to 0.672), 0.612 (95% CI: 0.553 to 0.670), 0.598 (95% CI: 0.539 to 0.657), and 0.630 (95% CI: 0.572 to 0.687), respectively, in the validation set. In the testing set, the AUC value of RF, LGBM, XGBoost, and LR, was 0.641 (95% CI: 0.516 to 0.766), 0.634 (95% CI: 0.511 to 0.758), 0.644 (95% CI: 0.521 to 0.768), and 0.645 (95% CI: 0.521 to 0.769), respectively. The AUC value of the bootstrap for RF, LGBM, XGBoost, and LR, was 0.705 (95% CI: 0.662 to 0.747), 0.670 (95% CI: 0.626 to 0.715), 0.789 (95% CI: 0.752 to 0.826), and 0.639 (95% CI: 0.593 to 0.684), respectively. The AUC, accuracy, sensitivity, specificity, PPV, and NPV of the predictive model for live birth before the first cycle are shown in Table 2.

**Table 2:** The predictive performance evaluation of the predictive models for live birth in women who received IVF or ICSI for the first time

Subgroups	Models	Datasets	AUC	Sensitivity (95% CI)	Specificity (95% CI)	PPV (95% CI)	NPV (95% CI)	Accuracy (95% CI)
Before the first cycle	RF	Train	0.721 (0.695–0.748)	0.740 (0.707–0.773)	0.602 (0.567–0.636)	0.620 (0.586–0.653)	0.725 (0.690–0.760)	0.666 (0.642–0.691)
Before the first cycle	RF	Valid	0.614 (0.555–0.672)	0.809 (0.747–0.872)	0.387 (0.320–0.454)	0.496 (0.434–0.558)	0.731 (0.648–0.815)	0.567 (0.516–0.619)
Before the first cycle	RF	Test	0.641 (0.516–0.766)	0.765 (0.622–0.907)	0.568 (0.422–0.715)	0.578 (0.433–0.722)	0.758 (0.611–0.904)	0.654 (0.548–0.759)
Before the first cycle	RF	Bootstrap	0.705 (0.662–0.747)	0.588 (0.528–0.649)	0.680 (0.627–0.732)	0.606 (0.545–0.667)	0.664 (0.611–0.716)	0.638 (0.599–0.678)
Before the first cycle	LGBM	Train	0.678 (0.651–0.706)	0.645 (0.609–0.681)	0.627 (0.592–0.661)	0.603 (0.567–0.638)	0.668 (0.633–0.703)	0.635 (0.610–0.660)
Before the first cycle	LGBM	Valid	0.612 (0.553–0.670)	0.678 (0.603–0.752)	0.539 (0.471–0.608)	0.523 (0.453–0.593)	0.692 (0.620–0.764)	0.598 (0.547–0.649)
Before the first cycle	LGBM	Test	0.634 (0.511–0.758)	0.824 (0.695–0.952)	0.477 (0.330–0.625)	0.549 (0.412–0.686)	0.778 (0.621–0.935)	0.628 (0.521–0.735)
Before the first cycle	LGBM	Bootstrap	0.670 (0.626–0.715)	0.602 (0.542–0.662)	0.636 (0.582–0.690)	0.581 (0.521–0.640)	0.657 (0.602–0.711)	0.621 (0.580–0.661)
Before the first cycle	XGBoost	Train	0.806 (0.784–0.829)	0.792 (0.762–0.823)	0.675 (0.642–0.709)	0.682 (0.649–0.715)	0.788 (0.756–0.819)	0.730 (0.707–0.753)
Before the first cycle	XGBoost	Valid	0.598 (0.539–0.657)	0.730 (0.660–0.801)	0.466 (0.397–0.534)	0.505 (0.438–0.571)	0.699 (0.621–0.776)	0.579 (0.527–0.630)
Before the first cycle	XGBoost	Test	0.644 (0.521–0.768)	0.824 (0.695–0.952)	0.523 (0.375–0.670)	0.571 (0.433–0.710)	0.793 (0.646–0.941)	0.654 (0.548–0.759)
Before the first cycle	XGBoost	Bootstrap	0.789 (0.752–0.826)	0.678 (0.621–0.736)	0.754 (0.705–0.802)	0.697 (0.640–0.755)	0.737 (0.688–0.786)	0.719 (0.682–0.757)
Before the first cycle	LR	Train	0.641 (0.613–0.670)	0.659 (0.623–0.695)	0.563 (0.528–0.599)	0.570 (0.535–0.605)	0.653 (0.616–0.689)	0.608 (0.583–0.633)
Before the first cycle	LR	Valid	0.630 (0.572–0.687)	0.539 (0.460–0.619)	0.672 (0.607–0.736)	0.550 (0.470–0.630)	0.662 (0.597–0.726)	0.615 (0.565–0.666)
Before the first cycle	LR	Test	0.645 (0.521–0.769)	0.765 (0.622–0.907)	0.568 (0.422–0.715)	0.578 (0.433–0.722)	0.758 (0.611–0.904)	0.654 (0.548–0.759)
Before the first cycle	LR	Bootstrap	0.639 (0.593–0.684)	0.505 (0.443–0.566)	0.679 (0.627–0.732)	0.568 (0.504–0.633)	0.621 (0.569–0.673)	0.600 (0.559–0.641)
Before and after the first cycle	RF	Train	0.871 (0.853–0.888)	0.913 (0.891–0.934)	0.661 (0.627–0.695)	0.703 (0.672–0.733)	0.896 (0.871–0.922)	0.779 (0.757–0.800)
Before and after the first cycle	RF	Valid	0.789 (0.742–0.836)	0.987 (0.969–1.000)	0.569 (0.501–0.637)	0.630 (0.569–0.692)	0.983 (0.960–1.000)	0.747 (0.702–0.792)
Before and after the first cycle	RF	Test	0.862 (0.781–0.943)	1.000 (1.000–1.000)	0.682 (0.544–0.819)	0.708 (0.580–0.837)	1.000 (1.000–1.000)	0.821 (0.735–0.906)
Before and after the first cycle	RF	Bootstrap	0.862 (0.833–0.892)	0.913 (0.879–0.948)	0.663 (0.610–0.716)	0.694 (0.644–0.743)	0.902 (0.863–0.941)	0.777 (0.742–0.811)
Before and after the first cycle	LGBM	Train	0.841 (0.821–0.861)	0.910 (0.888–0.932)	0.633 (0.599–0.668)	0.685 (0.655–0.716)	0.889 (0.862–0.915)	0.762 (0.740–0.785)
Before and after the first cycle	LGBM	Valid	0.816 (0.773–0.859)	0.993 (0.981–1.000)	0.554(0.486–0.622)	0.624 (0.563–0.685)	0.991 (0.974–1.000)	0.742 (0.696–0.787)
Before and after the first cycle	LGBM	Test	0.835 (0.743–0.926)	0.941 (0.862–1.000)	0.705 (0.570–0.839)	0.711 (0.579–0.844)	0.939(0.858–1.000)	0.808 (0.720–0.895)
Before and after the first cycle	LGBM	Bootstrap	0.839 (0.806–0.871)	0.917 (0.883–0.951)	0.627 (0.573–0.682)	0.673 (0.624–0.722)	0.901 (0.861–0.941)	0.759 (0.724–0.795)
Before and after the first cycle	XGBoost	Train	0.898 (0.883–0.914)	0.808 (0.778–0.837)	0.828 (0.802–0.855)	0.805 (0.775–0.835)	0.831 (0.804–0.857)	0.819 (0.799–0.839)
Before and after the first cycle	XGBoost	Valid	0.806 (0.761–0.851)	0.993 (0.981–1.000)	0.549 (0.481–0.617)	0.621 (0.560–0.682)	0.991 (0.974–1.000)	0.739 (0.693–0.784)
Before and after the first cycle	XGBoost	Test	0.843 (0.753–0.933)	1.000 (1.000–1.000)	0.682 (0.544–0.819)	0.708 (0.580–0.837)	1.000 (1.000–1.000)	0.821 (0.735–0.906)
Before and after the first cycle	XGBoost	Bootstrap	0.889 (0.863–0.915)	0.943 (0.915–0.972)	0.660(0.607–0.714)	0.699 (0.650–0.747)	0.933 (0.900–0.966)	0.789 (0.755–0.823)
Before and after the first cycle	LR	Train	0.656 (0.628–0.684)	0.753 (0.721–0.786)	0.479 (0.443–0.514)	0.559 (0.527–0.592)	0.689(0.649–0.728)	0.607 (0.582–0.633)
Before and after the first cycle	LR	Valid	0.652 (0.527–0.777)	0.882 (0.774–0.991)	0.455 (0.307–0.602)	0.556 (0.423–0.688)	0.833 (0.684–0.982)	0.641 (0.535–0.747)
Before and after the first cycle	LR	Test	0.668 (0.612–0.724)	0.789 (0.725–0.854)	0.525 (0.456–0.593)	0.553 (0.487–0.619)	0.770 (0.700–0.840)	0.638 (0.588–0.688)
Before and after the first cycle	LR	Bootstrap	0.665 (0.620–0.710)	0.499 (0.438–0.560)	0.722 (0.671–0.772)	0.600 (0.534–0.666)	0.633 (0.582–0.683)	0.620 (0.580–0.661)

*IVF* in vitro fertilization, *ICSI* intracytoplasmic sperm injection, *LR* logistic regression, *RF* random forest, *XGBoost* extreme gradient boosting, *LGBM* light gradient boosting machine, *NPV* negative predictive value, *PPV* positive predictive value, *AUC* area under curve, *CI* confidence interval

Four predictive models: LR, RF, LGBM, and XGBoost were also conducted based on the predictive factors before and after the first cycle. As Table 2 shows, the AUC value in the training set, validation set, testing set, and bootstrap of the RF was 0.871 (95% CI: 0.853 to 0.888), 0.789 (95% CI: 0.742 to 0.836), 0.862 (95% CI: 0.781 to 0.943), and 0.862 (95% CI: 0.833 to 0.892), respectively. The AUC value in the training set, validation set, testing set, and bootstrap of LGBM was 0.841 (95% CI: 0.821 to 0.861), 0.816 (95% CI: 0.773 to 0.859), 0.835 (95% CI: 0.743 to 0.926), and 0.839 (95% CI: 0.806 to 0.871), respectively. The AUC value in the training set, validation set, testing set, and bootstrap of XGBoost was 0.898 (95% CI: 0.883 to 0.914), 0.806 (95% CI: 0.761 to 0.851), 0.843 (95% CI: 0.753 to 0.933), and 0.889 (95% CI: 0.863 to 0.915), respectively. The AUC value in the training set, validation set, testing set, and bootstrap of LR was 0.656 (95% CI: 0.628 to 0.684), 0.652 (95% CI: 0.527 to 0.777), 0.668 (95% CI: 0.612 to 0.724), and 0.665 (95% CI: 0.620 to 0.710), respectively.

## Discussion

In this study, among the 1,857 women, 851 women (45.83%) ended up with live birth. We respectively constructed 8 machine learning models to predict the live birth for the first complete IVF attempt or ICSI using predictive factors before and after the first cycle. The result showed that the LGBM model achieved a robust performance before and after the first cycle compared with LR, XGB, and RF. LGBM showed better predictive performance on the live birth chance after the first cycle compared with the prediction models before the first cycle. The predictive factors before the first cycle included maternal age, AMH, basal FSH, basal P, basal E2, infertility duration, and the number of left sinus follicles. Sixteen predictive factors were identified that affect the live birth before and after the first cycle of IVF/ICSI including maternal age, AMH, basal E2, basal PRL, infertility duration, the number of pregnancies, the number of left sinus follicles, serum HCG level, EMT on HCG day, LH level on HCG day, E2 level on HCG day, Gn, method of fertilizations, types of transferred embryos, stimulation protocols, and endometrial preparation protocol.

Several predictive models have been developed to assess the outcome of IVF treatment. One of the early and most accepted prediction models is the McLernon model [16, 17], which utilizes only discrete LR to predict the chance of live birth for a couple. In addition, the McLernon model did not take several important factors such as BMI and AMH into account. Unlike classical statistics, machine learning algorithms can take into account complex associations between different parameters, and thus can better exploit the synergy between these correlated parameters [12]. ZoharBarnett-Itzhaki et al. [12] used age, BMI, and clinical characteristics to predict IVF outcomes in 136 women undergoing fresh IVF cycles. The author found that compared with LR, the accuracy of artificial neural network (NN) and support vector machine (SVM) was 0.69 to 0.9 and 0.45 to 0.77, respectively, while the accuracy was 0.34 to 0.74 using LR model, indicating that machine learning algorithms based on age, BMI, and clinical data are superior to LR in predicting IVF outcomes. CelineBlank et al. used the parameters before and after the cycle to develop a RF model to predict the implantation rate after fresh blastocyst transfer and compared it with the LR model [18]. The results showed that the AUC of the RF model was 0.74, and the AUC of the traditional LR model was 0.66. Qiu et al. used a machine learning method to establish a personalized prediction of live birth prior to the first IVF and found that the XGBoost model achieved an area under the ROC curve of 0.73. However, this model was only established based on the factors before the first cycle [7]. In this study, we used machine learning techniques to predict live birth in women undergoing IVF treatment or ICSI based on the predictive factors before and after the first cycle. Our results showed that the LGBM model had a robust performance before and after the first cycle of IVF treatment in comparison with LR. Our study also indicated that the prediction model indicated a better predictive performance on the live birth chance after the first cycle compared with the prediction models before the first cycle. We speculate that this possible reason may be that the prediction model based on the predictive factors after the first cycle is closer to the live birth outcome, so it is more suitable to predict the live births. This study might be a promising step to provide personalized estimates of live birth chance of the IVF or ICSI before and after the first cycle based on the machine learning algorithms.

In previous prediction models, female age was the most established predictive factor associated with live birth after IVF/ICSI [7, 12, 19]. A study by Hassan et al. proposed a hill-climbing feature selection algorithm with five different machine learning models to analyze and predict IVF pregnancies with higher accuracy and has found that age was the most important factor affecting IVF pregnancy outcomes [6]. Our study demonstrated that maternal age was related to live birth for women before and after the first cycle of IVF/ICSI. Female ovarian reserve gradually declines with age, and the quantity and quality of oocytes decrease significantly, which has a significant negative impact on the success of pregnancy in patients undergoing IVF/ICSI treatment [20]. We observed that the infertility duration, the number of pregnancies, and the number of left sinus follicles were associated with the birth rate in women with IVF treatment. Mo et al. found that the number of infertility years, and sinus follicles were related to the success of treatment of IVF-embryo transfer [21]. A prediction model has reported that the duration of infertility was the predictive factor of live birth prior to the first IVF treatment [7].

In this study, hormone levels could be the predictive factors for live birth in women with IVF or ICSI. AMH, basal FSH, basal P, and basal E2 were associated with live birth before the first cycle of IVF/ICSI and AMH, basal E2, basal PRL, serum HCG level, LH level on HCG day, E2 level on HCG day, and Gn were found to affect the live birth after the first cycle of IVF/ICSI. Gn has been used to induce multiple follicle development and increase ovarian stimulation efficiency in infertile women since the 1960s [22]. AMH can inhibit follicular proliferation and growth by limiting the functions of growth factors and Gn, and it directly reflects the condition of the primordial follicles and effectively predicts the patient's ovarian reserve [23]. A previous systematic review suggested that AMH has some value in the prediction of live birth, and can be a predictor of live birth in women undergoing assisted conception [24]. Estrogen levels in women would be reduced as ovarian reserve decreases, resulting in an increase in FSH secretion. The elevated basal FSH level is associated with the decline of ovarian reserve [25]. A study evaluating the impact of different etiologies of diminished ovarian reserve on pregnancy outcome in IVF-ET cycles indicated that elevated basal FSH affects the number of oocytes retrieved and the implantation rates after IVF/ICSI [26]. Farhi et al. reported that elevated E2 levels during IVF cycles were linked to a higher incidence of adverse pregnancy outcomes [27]. Lower basal E2 was associated with better pregnancy rates and ovarian reserve during IVF utilizing a gonadotropin releasing hormone (GnRH) antagonist [28]. Morales et al. reported serum E2 on the day of the trigger as a predictor of metaphase II oocytes in antagonist cycles encourage greater oocyte maturity and fertilization [29]. Chen et al. found that high serum E2 level on HCG day was associated with decreased live-birth rates in patients with frozen embryo transfer [30]. Previous studies showed that the alterations of these hormones such as basal LH, basal PRL, and basal P levels were associated with pregnancy in embryo transfer [31–33]. In addition to the hormone levels, a study [34] evaluating the probability of live birth after a freeze-all based on an IVF treatment strategy found that EMT was one of the most important predictive factors. A meta-analysis indicated that women with thinner EMT are inversely associated with IVF/ICSI success, whereas higher endometrium tends to confer the best outcome [35]. Wen et al. [19] have identified EMT as one of the independent predictors affecting IVF/ICSI success. EMT on HCG day was highlighted as a predictive factor for live birth in our model after the first cycle of IVF/ICSI.

In this study, the method of fertilization and types of transferred embryos were associated with live births in women who received IVF or ICSI. Previous studies indicated that the quality and implantation potential of frozen embryos is similar to or even better than that of fresh embryos [36–38]. In practice, the freeze-all strategy can reduce the risk of ovarian hyperstimulation syndrome (OHSS) in the ovarian stimulation cycle by avoiding pregnancy and obtaining better results [39]. However, the results of a previous meta-analysis showed no differences in fertilization rate, total fertilization failure rate, good embryo quality rate, fresh embryo implantation clinical pregnancy rate, fresh embryo transfer live birth rate, miscarriage rate, neonatal preterm birth rate and neonatal low birth weight rate in those treated with ICSI compared with IVF [40]. The relationship between the fertilization method and live birth needs further clarification. We found that stimulation protocols and endometrial preparation protocols were related to the live births in women who received IVF or ICSI. A previous study indicated that live birth rates per transferred embryo were significantly increased by GnRH agonist administration in women stimulated either with the long protocol or with a GnRH antagonist [41]. In women undergoing single euploid frozen blastocyst transfer, the natural cycles group had a lower pregnancy loss rate and ultimately a higher live birth rate compared with the hormone replacement treatment group [42]. A retrospective study of 12,950 frozen embryo transfer cycles published by Li et al. concluded a comparable clinical pregnancy rate and a lower live birth rate when hormone replacement treatment cycles were compared with natural cycles [42].

Our prediction models not only estimate the chances of success in couples before commencing IVF but also are able to revise these chances on the basis of the couple’s response to a first treatment cycle. The prediction model before the first cycle can be used to interrogate couples before treatment, while the predictive model after the first cycle predicts the chances of future success for couples considering further treatment. Our study has a number of strengths. First of all, we introduced the parameters before and after the first cycle when constructing the models for live birth, which provided the summary information of the success opportunities before and after the treatment. Secondly, machine learning methods were used to predict live birth in pregnant women undergoing the first IVF in the Chinese population, including fresh and frozen embryo transfer. Thirdly, the factors included are comprehensive, which improves the prediction ability of the prediction models. However, several limitations deserve to be mentioned. Firstly, the study was retrospective, single-center, and encompassed a limited number of patients. Secondly, the lack of external validation and the study population was pregnant women undergoing IVF for the first time, which may limit the popularization of our research results. Thirdly, without an impact analysis, it is impossible to verify whether the clinical decisions made by clinicians combined with the prediction performance of the models can improve the live birth rate or reduce the treatment cost. Finally, the prediction models were constructed based on limited predictive factors obtained before and after IVF treatment, however, pregnancy is a dynamic and ongoing process, and there are many other confounders that have an effect at different time points.

## Conclusion

This study indicated that machine learning-based predictive model could be used as a tool to predict live birth in women with IVF cycles, This study may help predict the chances of live birth before and after the first cycles of IVF or ICSI using personalized information, helping shape couples’ expectations of their IVF or ICSI outcome, allowing them to plan their treatments more efficiently and prepare emotionally and financially.

## Data Availability

The datasets used and/or analyzed during the current study are available from the corresponding author on reasonable request.
